# Gliadin-mediated green preparation of hybrid zinc oxide nanospheres with antibacterial activity and low toxicity

**DOI:** 10.1038/s41598-021-89813-0

**Published:** 2021-05-14

**Authors:** Qun Wang, Peng Ji, Yansheng Yao, Yi Liu, Yajie Zhang, Xianglong Wang, Yuhang Wang, Jinyan Wu

**Affiliations:** 1 College of Pharmacy and Chemistry & Chemical Engineering, Jiangsu Provincial Key Laboratory of Chiral Pharmaceutical Chemicals Biologically Manufacturing, Taizhou University, Taizhou, 225300 Jiangsu China; 2 Department of Endocrinology, The Affiliated Taixing People’s Hospital of Medical College, Yangzhou University, Taixing, 225400 China; 3 Department of Dermatology, Huashan Hospital, Fudan University, Jing’an District, Shanghai, 200040 China

**Keywords:** Pharmaceutics, Antimicrobial therapy, Nanomedicine, Nanoparticles, Drug safety

## Abstract

The development of inorganic antibacterial agents that impart antibacterial properties to biomaterials has attracted wide attention. The paper introduced a kind of hybrid nanosphere antibacterial agent composed of wheat gliadin (WG) and zinc oxide (ZnO), with antibacterial efficacy and low toxicity. The ZnO/WG hybrid nanospheres were environment-friendly integrated by molecular self-assembly co-precipitating and freeze-drying transformation, and were characterized using X-ray diffraction (XRD), Fourier transform infrared spectroscopy (FTIR), scanning electron microscope (SEM), atomic absorption spectroscopy (AAS), specific surface and pore size analysis, bacteriostasis test, reactive oxygen species (ROS) determination and safety evaluation. It was found that the prepared hybrid nanospheres were composed of two components, WG and ZnO, with a diameter scope of 100–200 nm; the content of ZnO in the hybrid nanospheres can reach 46.9–70.2% (w/w); the bacteriostasis tests proved that the prepared ZnO/WG nanospheres generating ROS, have a significant inhibitory effect on *E. coli* and *S. aureus*; furthermore, the ZnO/WG nanospheres are relatively safe and highly biocompatible in cells and mice. Therefore, the prepared novel ZnO/WG hybrid nanospheres were supposed to apply in the preparation of anti-infective wound dressings, tissue engineering skin scaffold materials, food, and cosmetics preservatives, and so on.

## Introduction

Metal-based drugs, especially inorganic nanomaterials used in implantable biomaterials, have been studied as a next-generation nanomedicine^1,2^. However, implanted biomaterials, long or short periods of close contact with human tissue, have the problem of causing various bacterial infections^3–7^. Therefore, the improvement of the antibacterial properties of biomaterials has become the key to their wider application in clinical medicine.

It is one of the methods to improve the antimicrobial properties of biomaterials by inserting antimicrobial substances into biomaterials. However, when loaded into biomaterials, the antibiotics will be quickly exhausted, and this approach can easily make bacteria resistant; besides, although the antibacterial effect of quaternary ammonium salts is better, the application is also limited, due to the hemolytic effect^8^.

Inorganic antibacterial agents are durable and less toxic^9^. Metal or metal oxide nanoparticles are currently the main research directions of inorganic antibacterial agents^10–12^, especially research concerning microwaveocaloric therapy, sonodynamic treatment, and photoactivated therapy as effective and rapid antibacterial methods has attracted tremendous interest^13^. Nano-ZnO has attracted extensive attention due to its advantages, such as long-term antibacterial, safety^14^, stability, excellent angiogenesis, and promotion of proliferation of adult dermal fibroblast cells^15–17^.

Composite nanomaterials, obtained by integrating organic and inorganic components, have broad application prospects in many fields. Notably, these nanocomposite particles not only inherited the superior characteristics of the raw materials, but also obtained other multi-functional characteristics that are not available in a single component. For example, a growing number of researchers pay attention to the design of core–shell structured composite nanospheres^18,19^, which can reduce their toxicity by covering other materials^20–22^, and increase their biocompatibility and water dispersibility^23–25^.

In this paper, a hybrid nanosphere of inorganic antimicrobial ZnO and WG was prepared by self-assembly co-precipitating and freeze-drying transformation. This study is aimed to develop an anti-infective pharmaceutical for surgical trauma therapy and to develop a new tissue-engineered skin with persistent anti-infective properties^26–28^.

## Results and discussion

### Preparation of ZnO/WG nanospheres

**Figure 1:**
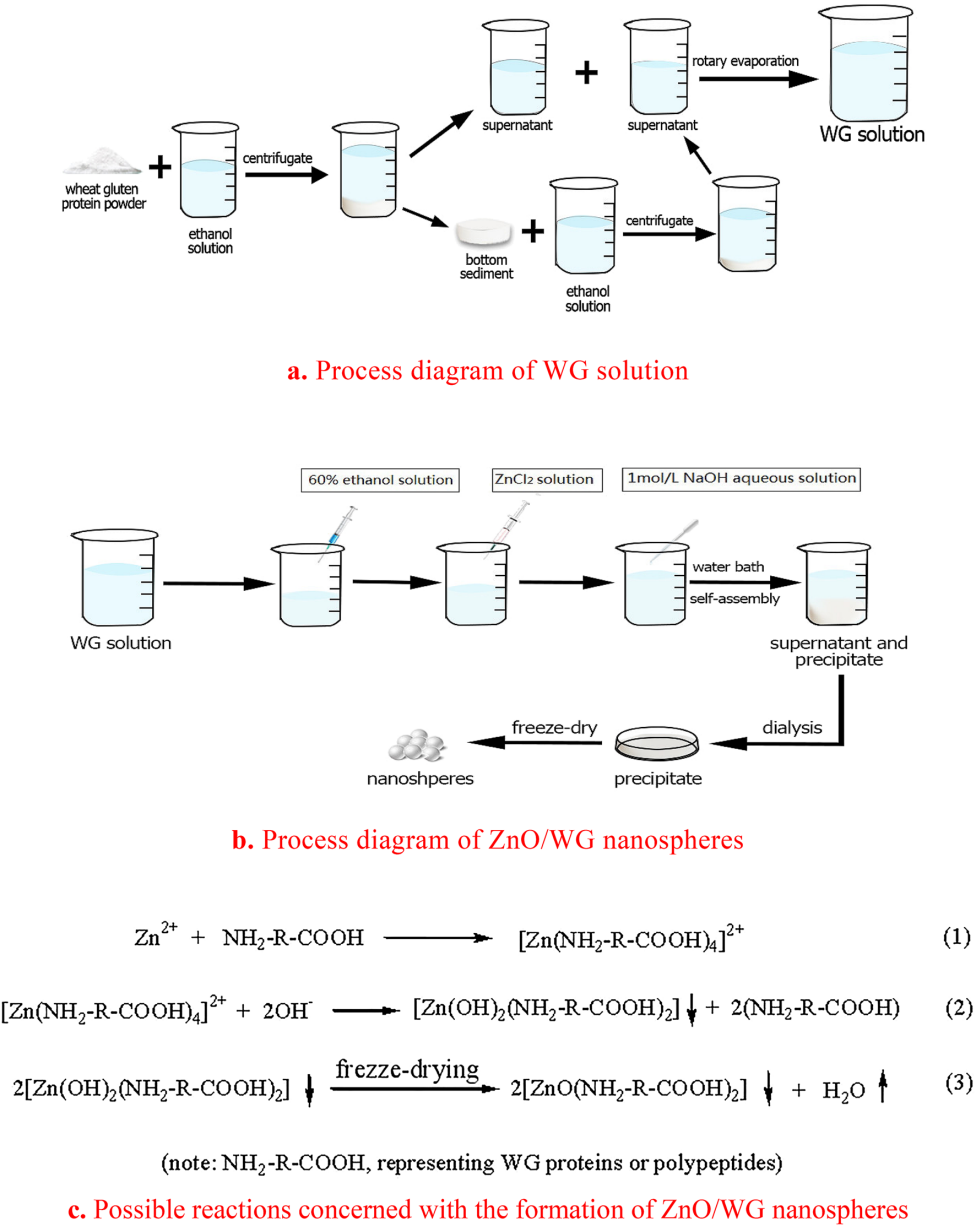
Preparation process (**a**,**b**) and possible reaction mechanism (**c**).

Generally speaking, the most common synthesis methods of ZnO nanoparticles were in the presence of surfactants or precipitating agents being associated with potential toxic effects against the environment and living orgnisms^29^. Attempting to address this issue, the preparation process of ZnO/WG nanospheres, as illustrated in Fig. 1a,b, was carried out in an environment-friendly manner, avoiding extreme reaction conditions, harmful chemicals, and toxic by-products, which would contribute better biocompatibility to the final applications of the nanospheres. The WG molecules here used, rich in amino, carboxyl, and hydroxyl groups, are stabilizing agents triggering the self-assembly co-precipitating of hybrid nanoparticles.

Substantially, according to molecular orbital theory, Zn^2+^ has four empty orbitals and are SP^3^ hybrids, so Zn^2+^ are easy to form tetra-ligand compounds when there are enough ligands as amine ligand et al. Therefore, the possible formation mechanism of ZnO/WG nanospheres is illustrated as follows:

The formation process of ZnO/WG nanospheres was shown in the above three formulations (Fig. 1c). Firstly, complexes were self-assembled formed by Zn^2+^ and WG polypeptides; secondly, two hydroxyl groups substituted two NH_2_-R-COOH ligands, which resulted in the co-precipitating formation of zinc hydroxic conjugating with WG polypeptides; finally, the co-precipitates lost water and transformed to the ZnO/WG nanospheres during the freeze-drying process.

Actually, the transformation between the zinc hydroxic conjugates and ZnO/WG nanospheres should be confirmed by the XRD test and FTIR test, as analyzed below.

### XRD and FTIR analysis

**Figure 2:**
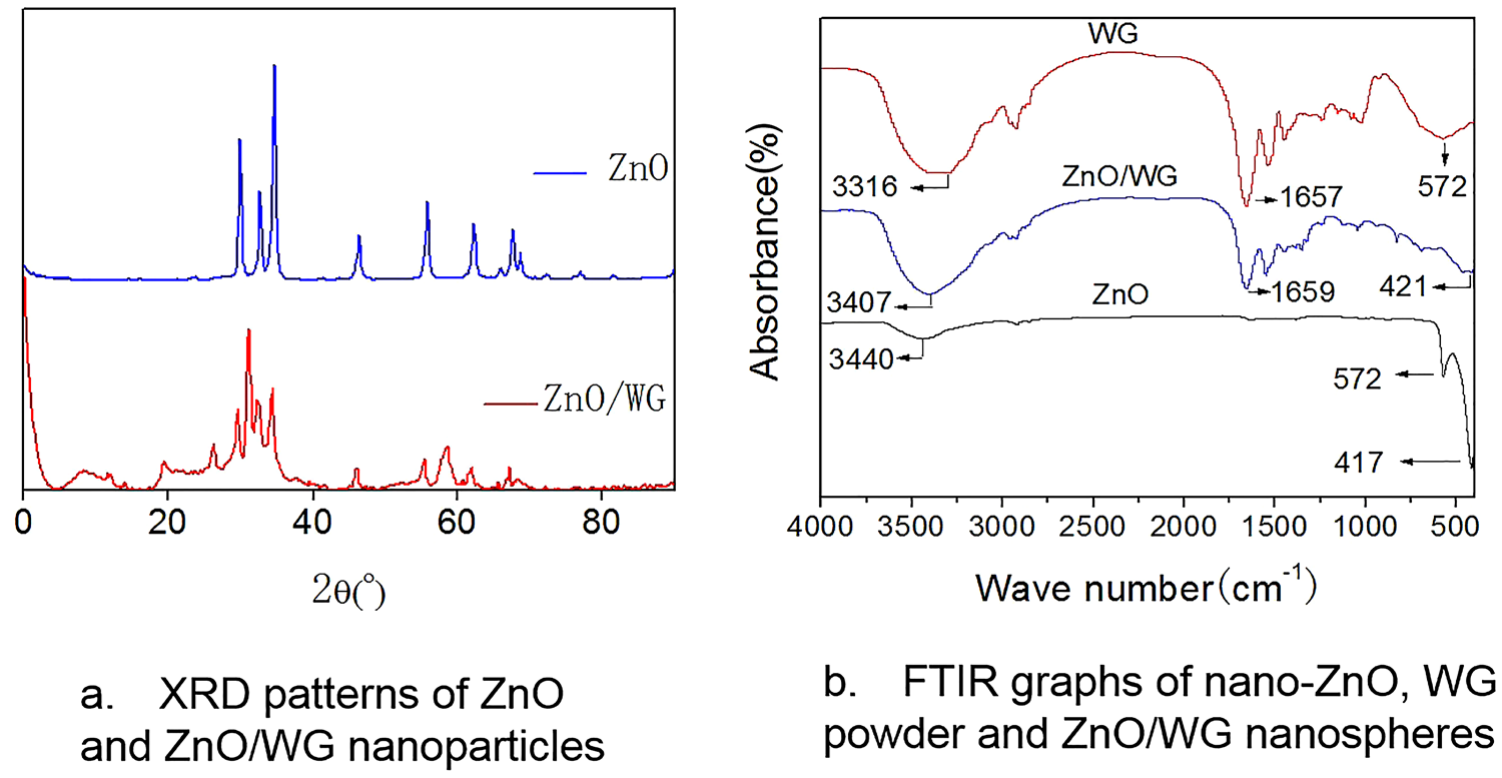
XRD patterns (**a**) and FTIR graphs (**b**).

As shown in Fig. 2a, due to the presence of ZnO crystals in the sample, the XRD pattern of the nano-ZnO has nine characteristic peaks, including 31.8°, 34.5°, 36.4°, 47.8°, 56.8°, 62.9°, 66.5°, 68.0°, 69.1°. And same peaks also appeared in ZnO/WG nanospheres, including 31.8°, 34.5°, 36.4°, 47.8°, 56.8°, 62.9°, 66.5°, 68.0°, 69.1°, indicating that ZnO crystals also exist in the ZnO/WG nanospheres^30,31^. Furthermore, in the XRD patterns of the ZnO/WG sample, peaks appeared at 8.28°, 19.5°, 33.2°, 58.6°, different from the characteristic peaks of ZnO, were possibly due to the two-dimensional lattice of zinc basic salt, a kind of nano-hybrid formed by the interaction between Zn^2+^ and WG molecules^32^.

In Fig. 2b, 3000–3600 cm^−1^ is the composite peak formed by the O–H stretching vibration of the -COOH group and the N–H stretching vibration of the –NH_2_ group; peaks near 1659 cm^−1^ are the C=O stretching vibration peak of the amide bond, peaks near 1540 cm^−1^ are formed by the N–H stretching vibration of the amide bond, peaks near 1450 cm^−1^ are formed by the C–N stretching of the amide bond; these peaks are typical characteristic peaks of proteins. Furthermore, due to the stretching vibration of Zn–O, a peak near 417 cm^−1^ appeared the characteristic peak of ZnO. Therefore, according to the comparison among the three FTIR graphs, it was proved that the ZnO/WG nanospheres were composed of ZnO and WG, which is consistent with the XRD analysis results.

### Morphology observation and analysis of ZnO/WG nanospheres

**Figure 3:**
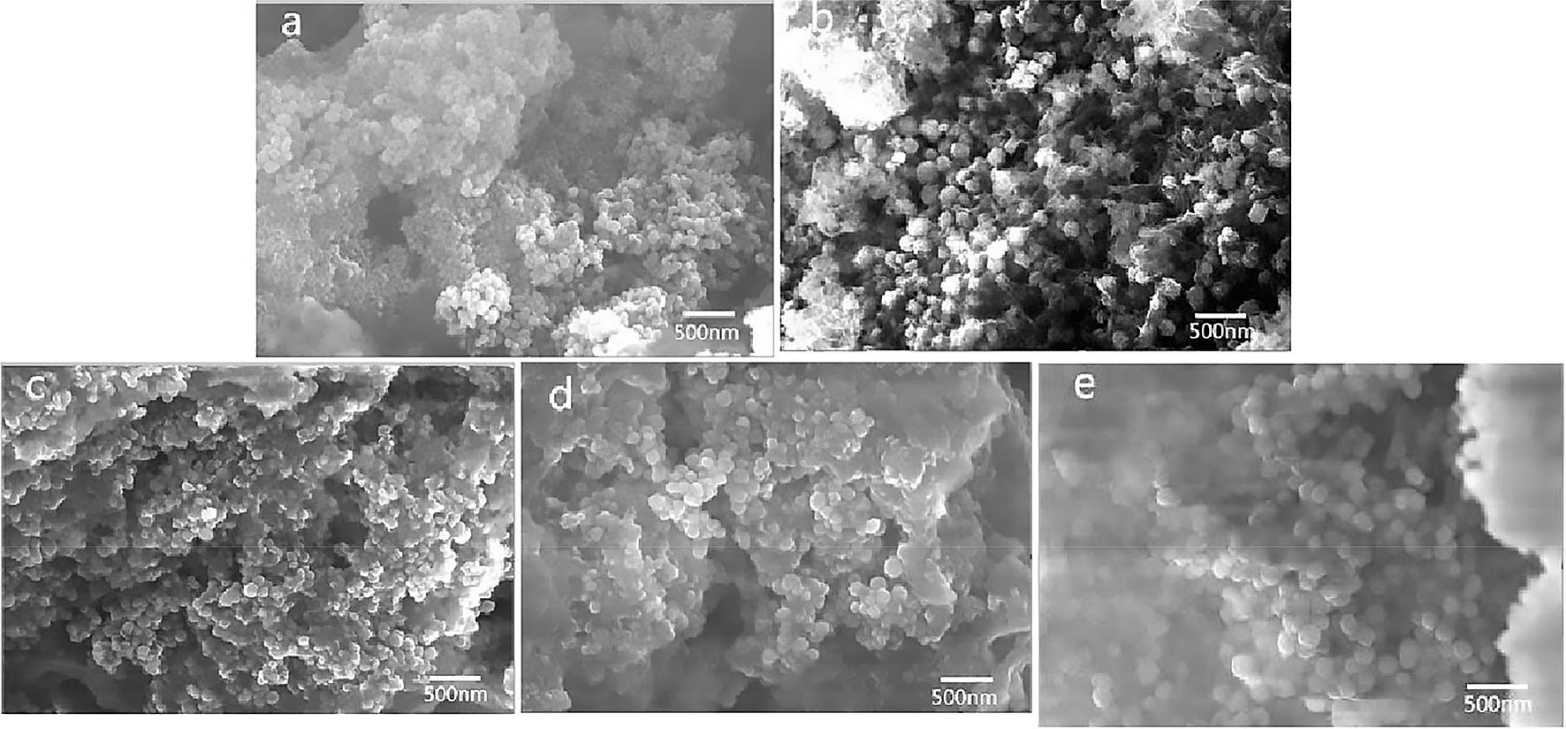
ZnO/WG nanospheres prepared by different concentrations of WG [WG concentrations while preparation: (**a**) 2 mg/mL; (**b**) 4 mg/mL; (**c**) 6 mg/mL; (**d**) 8 mg/mL; (**e**) 10 mg/mL].

The SEM images of ZnO/WG nanospheres were shown in Fig. 3. When the WG concentration in the preparation process is 2 mg/mL, 6 mg/mL, 8 mg/mL, 10 mg/mL, the nanospheres are spherical or ellipsoidal, with no holes on the surface, but there is a certain amount of adhesion between the particles; when the WG concentration is 4 mg/mL, the shape of the prepared nanospheres appeared a more irregular spherical shape with rough surface, but the independence between the particles is better. In conclusion, the common profiles of the prepared hybrid nanospheres were revealed: the shape is nearly spherical, the particle size range is 100–200 nm, but the adhesion between the particles is relatively serious. The separation process between the supernatant and precipitates, as well as the freeze-drying method, might have a great influence on the adhesion of ZnO/WG nanospheres, which should be further researched in the following work.

**Figure 4:**
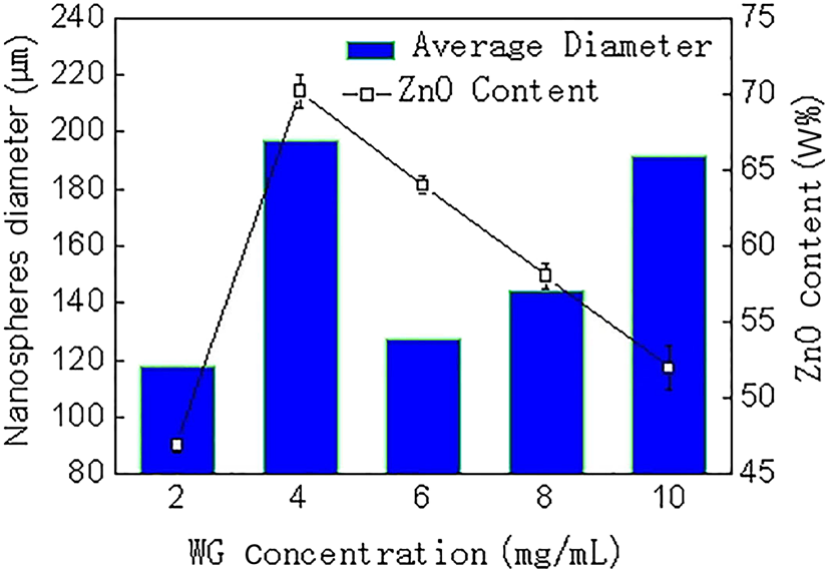
Effect of WG concentrations on the diameter and ZnO content of nanospheres.

According to the above observation of morphology, the particle size changed with the WG concentration increasing. Furthermore, as shown in Fig. 4, the ZnO content of the five different samples varied with the concentration of WG used in the preparation process, and its range reached 46.9–70.2% (w/w). Especially, when the WG concentration was 4 mg/mL, the average particle size of the corresponding nanospheres reached a maximum value of 197 nm, and the ZnO content also reached a maximum value of 70.2% (w/w). Therefore, the particles size and the content of the ZnO were functions of the concentration of WG.

### Specific surface area and pore size analysis

**Figure 5:**
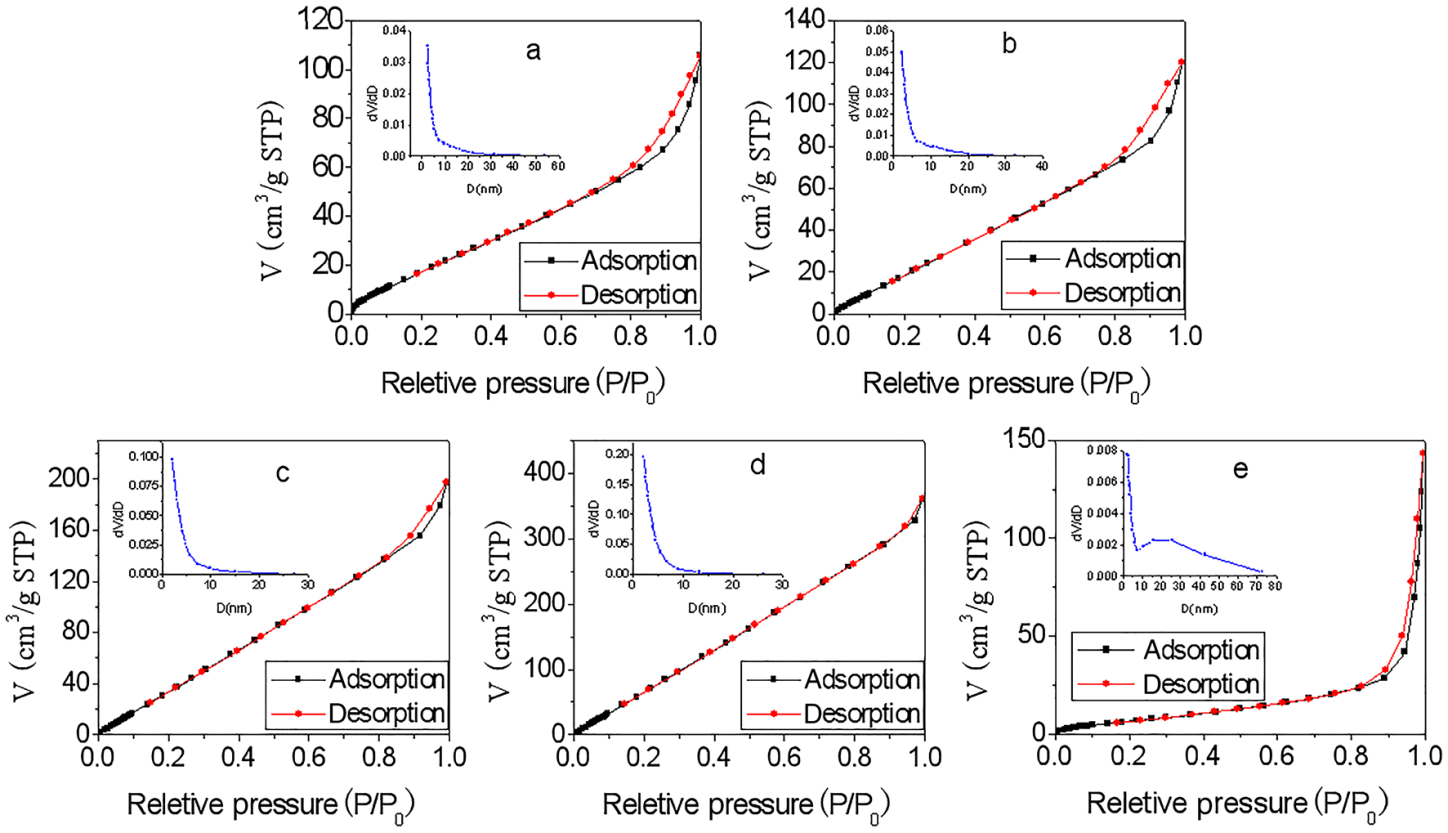
Isothermal adsorption and desorption curves (N_2_) and pore size distributions (BJH desorption) [WG concentrations while preparation: (**a**) 2 mg/mL; (**b**) 4 mg/mL; (**c**) 6 mg/mL; (**d**) 8 mg/mL; (**e**) 10 mg/mL].

As shown from the isothermal adsorption and desorption curves in Fig. 5, there is a ring caused by desorption lag at the relative pressure 0.8–1.0 which is a feature of mesoporous materials, indicating that the prepared ZnO/WG hybrid nanospheres are porous structures. According to the pore size distribution curves measured by the BJH method, the pore size distribution of samples a, b, c, and d ranged from 2 to 30 nm which was typical of mesoporous materials, and mainly distributed in the narrow range of 2–10 nm indicating a good consistency. However, the pore size distribution of sample e was wide, with different characteristics at 2–7 nm and 7–70 nm, respectively indicating poor pore size homogeneity, which may be caused by more WG protein macromolecules in the nanospheres.

Table 1 shows the most frequent pore size (BJH), specific surface area (BET), and pore volume of the 5 groups of samples. As the concentration of WG increasing from 2 to 10 mg/mL, the BJH desorption most frequent pore diameter of the prepared ZnO/WG hybrid nanospheres becomes firstly smaller (a,b,c,d) and lastly bigger (e), and respectively, the BET specific surface area and BJH desorption pore (D:1.7–300 nm) volume become firstly larger (a,b,c,d) and lastly smaller (e). According to the value of micropore volume measured by the HK method, there were small volumes of micropores in all 5 samples, but they accounted for a small proportion of the total volume, which was consistent with the results of pore size distribution curves in Fig. 5.

**Table 1:** The data concerned with specific surface area and pore size.

Sample	BET specific surface area(m^2^/g)	BJH Desorption Most Frequent Pore Diameter ((dV/dD)/nm)	BJH Desorption Pore (D:1.7–300 nm) Volume(cm^3^/g)	HK Micropore (D < 2 nm) Volume(cm^3^/g)
a	52.795	2.308	0.191	0.022
b	70.963	2.223	0.234	0.021
c	215.180	2.149	0.402	0.037
d	523.886	2.127	0.753	0.070
e	21.669	2.215	0.232	0.008

WG concentrations while preparation: (a) 2 mg/mL; (b) 4 mg/mL; (c) 6 mg/mL; (d) 8 mg/mL; (e) 10 mg/mL.

### Analysis of antibacterial test results

**Figure 6:**
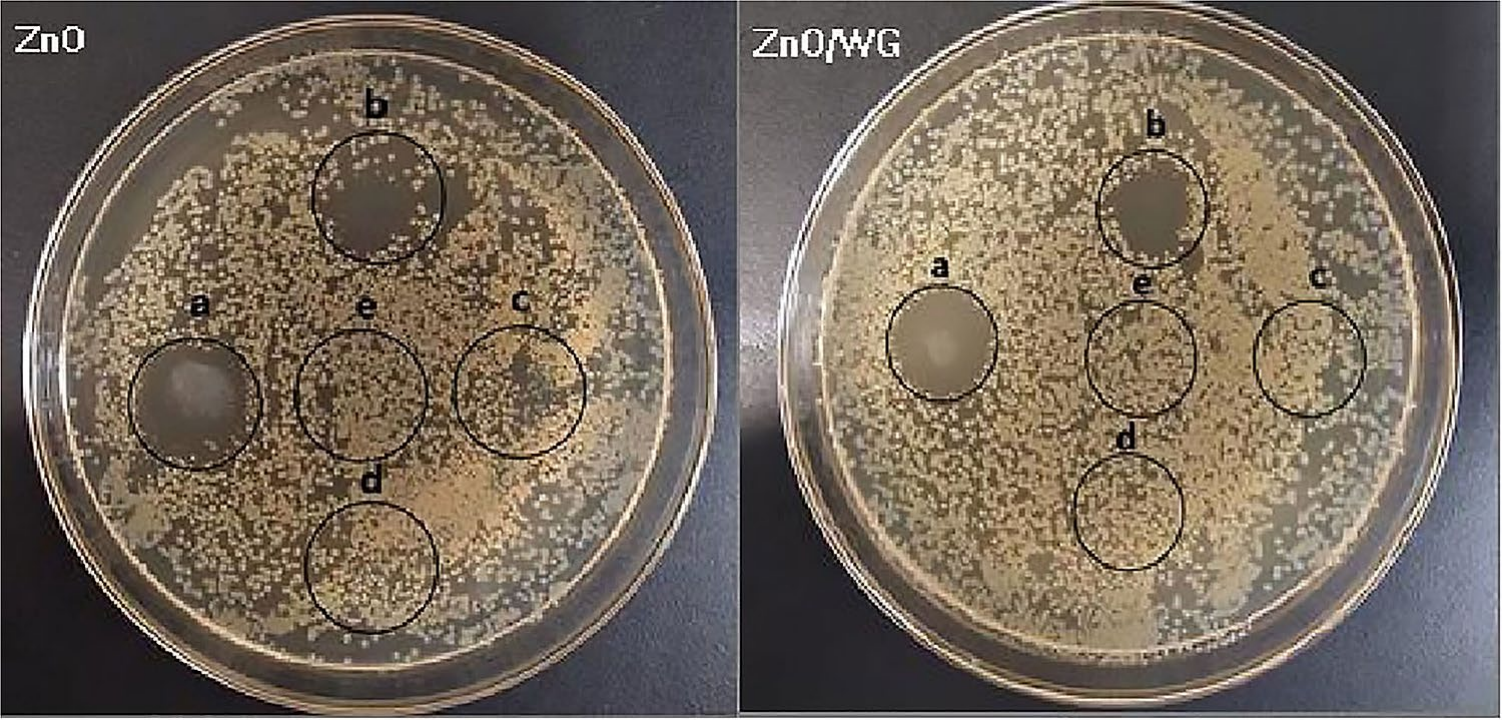
Representative sample agar plates showing the zones of inhibition to *S. aureus* (Suspension concentrations of samples: (**a**) 10 mg/mL; (**b**) 5 mg/mL; (**c**) 2 mg/mL; (**d**) 1 mg/mL; (**e**) saline).

As shown in Fig. 6, the antibacterial effect of the prepared hybrid nanospheres is represented by a circular sterile area. When the same volume of medicine (25 μL per sample) with different concentrations, was dripped onto the agar plate, the inhibitory effect of the nanoparticles on microorganisms can be indirectly tested depending on the concentration change of the suspension. The detailed results were given in Table 1 and discussed as follows.

Taking ZnO/WG hybrid nanospheres with a ZnO content of 70% (w/w) as an example, it can be found from Table 2: Negative control (saline) has no bacteriostatic activity; positive control (ZnO nanopowder), at a concentration of 2 mg/mL, has a certain bacteriostatic effect on *E. coli* and *S. aureus*; ZnO/WG hybrid nanospheres, at a concentration of 5 mg/mL, have a certain bacteriostatic effect on *E. coli* and *S. aureus*. At a concentration of 10 mg/mL, Both the hybrid nanospheres and the ZnO nanopowder have an obvious antibacterial effect on *E. coli* and *S. aureus*. This shows that the prepared ZnO/WG hybrid nanospheres have antibacterial activity increasing with the concentration^33,34^.

**Table 2:** Antibacterial effect of ZnO/WG nanospheres on *E. coli* and *S. aureus.*

Sample	Saline	ZnO nano-power suspension concentration (mg/mL)				The concentration of ZnO/WG hybrid nanosphere suspension (mg/mL)			
		10	5	2	1	10	5	2	1
*E. coli*	−	++	++	+ +	−	++	+	−	−
*S.aureus*	−	+++	++	+	−	+++	++	−	−
Antibacterial effect	×	√	√	√	×	√	√	×	×

While D ≥ 10 mm, and no bacteria colony in the inhibition zone, recorded as +++; while D ≥ 10 mm, but appeared visible colonies in the inhibition zone, recorded as ++; while 10 mm > D ≥ 5 mm, and no colony in the inhibition zone, it is recorded as ++; while 10 mm > D ≥ 5 mm, but appeared visible colonies in the circle, it is recorded as +; while D < 5 mm, recorded as −.

Besides, compared with ZnO nano-powder, at the low concentration, 2 mg/mL and 5 mg/mL, the bacteriostatic effect of ZnO/WG hybrid nanospheres on *E. coli* reduced; but at the concentration of 10 mg/mL, the antibacterial activity on *S. aureus* is equivalent. This shows that: Increasing the concentration of ZnO/WG hybrid nanospheres, can produce the equivalent bacteriostatic effect as the ZnO nano-power, which is beneficial to the application of ZnO/WG hybrid nanospheres, as a candidate antibacterial agents, in the fields of antibacterial biomaterials, food, and cosmetics preservatives, etc^35,36^.

### Antimicrobial mechanism

**Figure 7:**
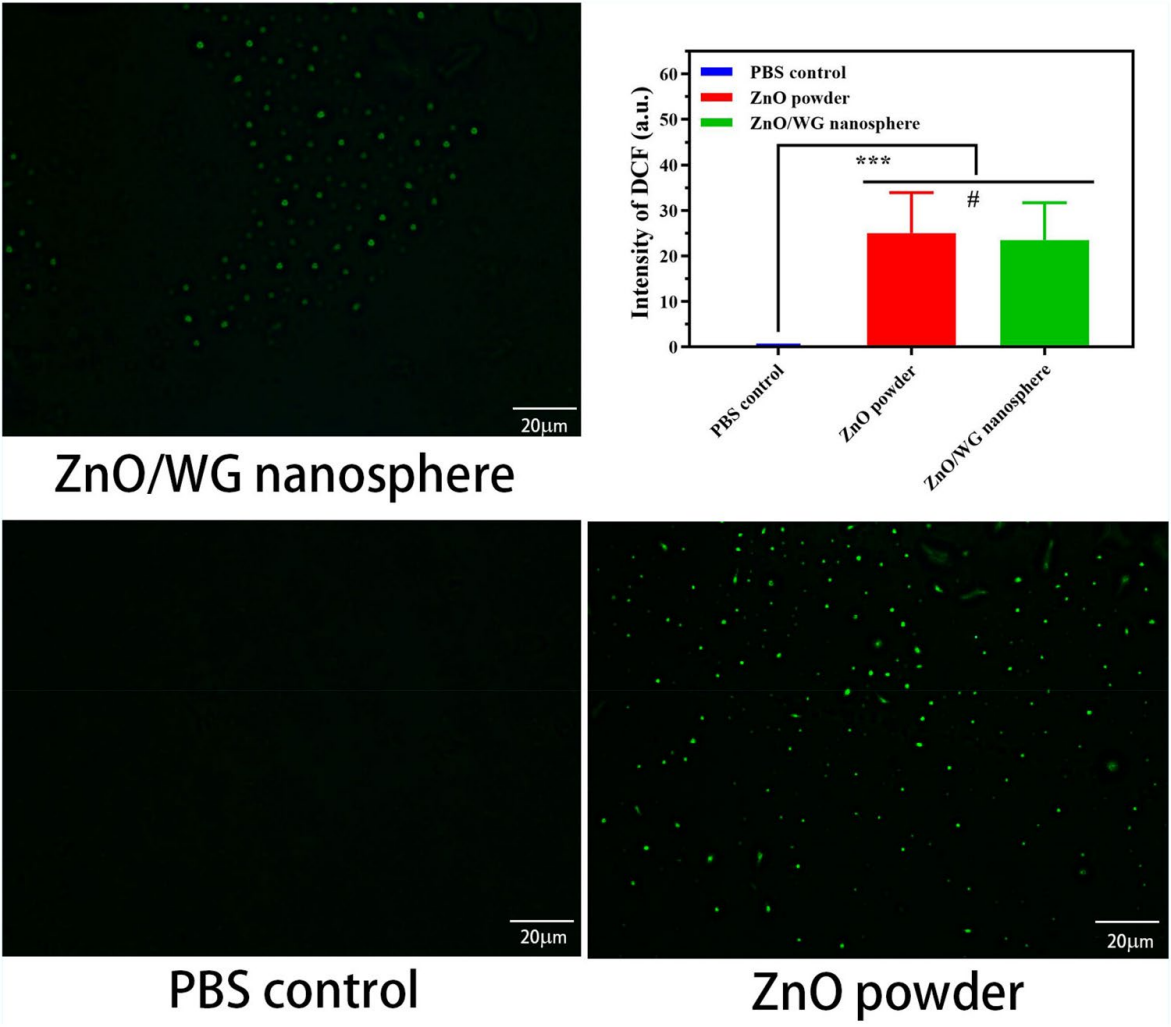
The results of ROS determination (Scale bar:20 μm, Results are expressed as the mean ± SD; corresponding DCF fluorescence value ***p < 0.005, ^**#**^*p* > 0.5).

A ROS determination test as shown in Fig. 7, was carried out to research the antibacterial mechanism. From the fluorescent probe photos, it was found that there were no fluorescent spots in PBS negative control group, while a large number of fluorescent spots appeared in samples of ZnO nanopowder and ZnO/WG hybrid microspheres which appeared stronger intensity of the intracellular ROS signal than that of PBS for *S. aureus* (*p* < 0.005). It was due to the intracellular ROS stimulating the conversion of DCFH-DA to a green fluorescent product (DCF)^37^.

Photoresponsive antimicrobial materials combat pathogenic bacteria via ROS, which is a general term describing the chemical species formed by incomplete oxygen reduction. ROS mainly includes superoxide anion (·O_2_), hydrogen peroxide (H_2_O_2_), singlet oxygen (^1^O_2_), and hydroxyl radical (·OH). It is widely accepted that ROS can bind to and damage bacterial membranes and cell walls, thereby destroying the bacterial defense system. ROS can also penetrate bacterial membranes, entering the cells to destroy proteins and lipids and, thus, directly or indirectly disrupting cellular respiration and other physiological activities^38–40^.

Furthermore, it has been reported that Zn^2+^ has an excellent antibacterial effect and can enhance oxidative stress and combine with the bacteria to change the fluidity of the bacterial membrane^41^. Nevertheless, as shown in Fig. 6, the method of the antimicrobial test was a bacteriostatic circle, therefore there was no appropriate acidic micro-environment for quickly releasing zinc ions converted from ZnO, and the nanospheres and bacteria stayed on the solid agar plate, had difficulty directly contact with each other, which resulted in weak interaction between the nanospheres and *S. aureus*.

Consequently, the antimicrobial properties of the prepared hybrid nanospheres might not be directly attributed to the release of zinc ions, as well as the interaction of the nanospheres and bacteria, but be mainly attributed to the ROS which moved quickly to contact with bacteria, and then damaged bacterial membranes and cell walls.

### Analysis of safety evaluation

**Figure 8:**
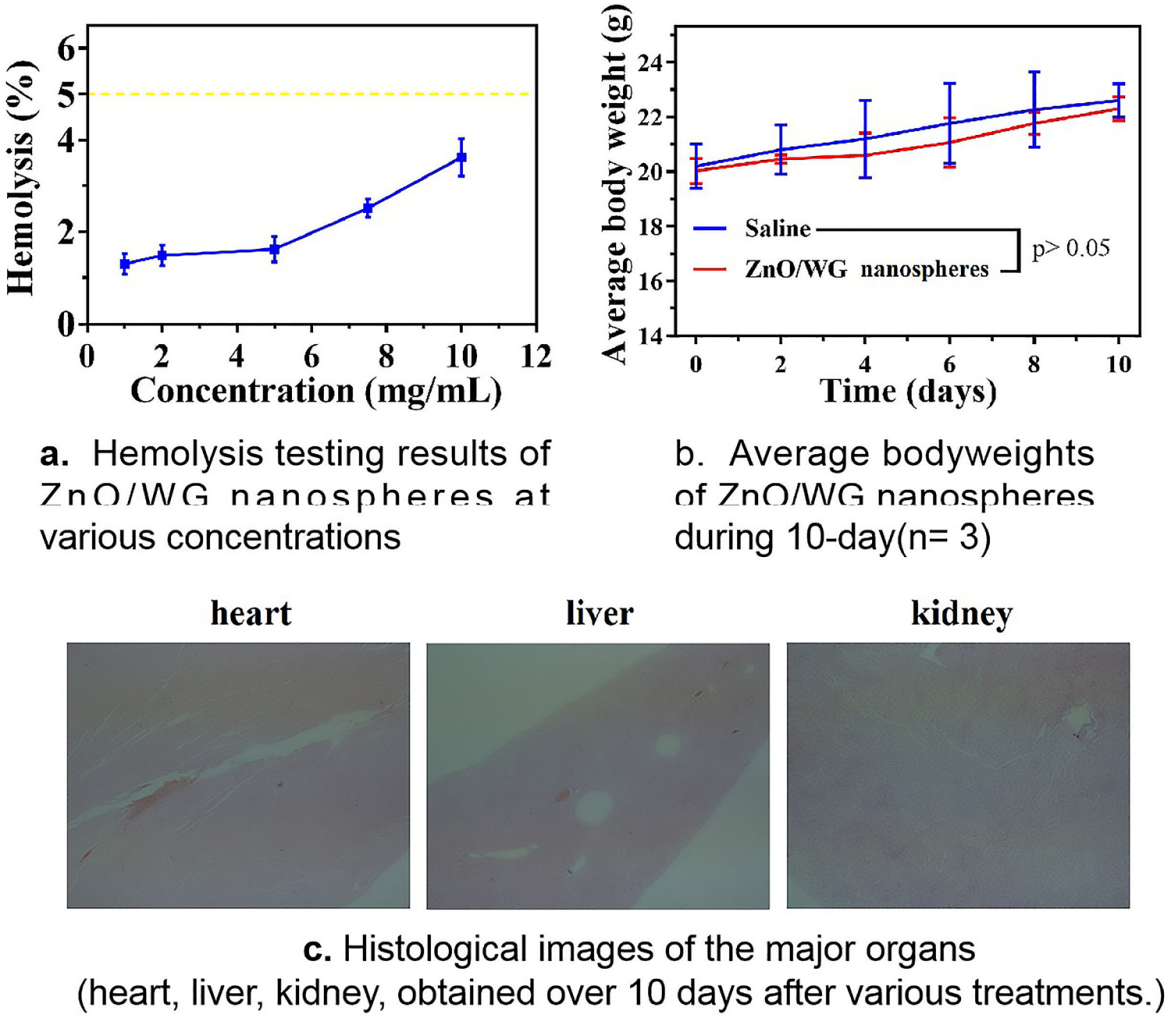
Safety evaluation.

To further evaluate the potential of ZnO/WG nanospheres in biomedical applications, the biocompatibility of the ZnO/WG nanospheres in vivo was examined in a mouse model. The breakdown of red blood cells collected from the mice was negligible for ZnO/WG nanospheres up to 10 mg/mL (Fig. 8a). For preparation, no significant weight loss was observed in the mice throughout the trial period (Fig. 8b), and the retention of ZnO/WG nanospheres in the heart, liver, and kidneys did not cause severe side effects, according to H&E staining (Fig. 8c), elucidating no serious adverse effects and elucidating good biocompatibility of ZnO/WG nanospheres in vivo^42^. These results generally indicated that the administration of ZnO/WG nanospheres was biocompatible in mice^43,44^.

## Conclusion

Generally, ZnO/WG nanospheres were prepared by zinc chloride solution using the self-assembly–freeze-drying method, under the induction of WG, at a suitable pH value.

Based on the XRD and FTIR analysis, it was confirmed that the nanosphere was composed of ZnO and WG. Based on the SEM test, it was observed that the shapes of ZnO/WG nanospheres were nearly spherical, with a diameter range of 100–200 nm. According to AAS data, it was determined that the content of ZnO in the nanospheres reaches 46.9–70.2% (w/w). The bacteriostasis tests proved that the prepared ZnO/WG nanospheres generating ROS, have a significant inhibitory effect on *E. coli* and *S. aureus*. Moreover, the hemolysis assays and H&E staining demonstrated that ZnO/WG nanospheres had relatively low toxicity in cells and mice.

## Materials and methods

### Materials

Gluten protein powder (5000 Da, food-grade, Jiangsu Zhihui Biotechnology Co., Ltd., China); zinc chloride (ZnCl_2_), ethanol, sodium hydroxide (NaOH), acetic acid (analytically pure, Sinopharm Group Chemical Reagent Co., Ltd., China); Zinc (99.99%, premium-grade, Sinopharm Group Chemical Reagent Co., Ltd., China); Concentrated hydrochloric acid (analytical grade, Wuxi City Yasheng Chemical Co., Ltd., China), ROS detection kit (Beijing Bio Lebo Technology Co., Ltd, China).

Female Balb/c mice (5–6 weeks old) were obtained from the Laboratory Animal Center of China Pharmaceutical University. All animal procedures were performed following the Guidelines for the Care and Use of Laboratory Animals of Taizhou University and approved by the Animal Ethics Committee of Taizhou University.

FTIR-650 Fourier Transform Infrared Spectrometer (FTIR) (FTIR-650, Tianjin Gangdong Technology Co., Ltd., China); Ultima IV X-ray Diffraction Analyzer (XRD) (Ultima IV, Japan Rigaku Co., Ltd., Japan); Sigma-500 Field Emission Scanning Electron Microscope (SEM) (Sigma-500, Carl Zeiss, Germany); SCIENTZ-10 N freeze dryer (Ningbo Xinzhi Biotechnology Co., Ltd., China); TAS-990AFG atomic absorption spectrophotometer (AAS) (Beijing Purkinje General Instrument Co., Ltd., China); Inverted fluorescence microscope (OLYMPUS IX51, Japan); Specific surface and pore size analyzer (JW-BK100).

### Preparation methods

#### Preparation of wheat gliadin (WG) solution

The process diagram of wheat gliadin (WG) solution is shown in Fig. 1a. About 4 g wheat gluten protein powder was slowly poured into 200 mL 70% ethanol solution after stirring for 2 h, and centrifuged to obtain supernatant and precipitate; bottom sediment was poured into 200 mL 70% ethanol solution under stirring. In the medium, stirring was continued for another 2 h, and the supernatant was centrifuged and combined with the previous supernatant, and concentrated by rotary evaporation to obtain a WG solution with a concentration of 1% (w/w).

#### Preparation of ZnO/WG nanospheres

The process diagram of ZnO/WG nanospheres is shown in Fig. 1b. Firstly, the above prepared solution of WG, was filled in 5 beakers, each 50.00 mL respectively, in which 200.00 mL, 75.00 mL, 33.50 mL, 12.50 mL, and 0 mL of 60% ethanol were added subsequently, to obtain five kinds of WG solutions with different concentrations of 2 mg/mL, 4 mg/mL, 6 mg/mL, 8 mg/mL, 10 mg/mL. And then 125.00 mL, 62.50 mL, 41.75 mL, 31.25 mL, 25.00 mL solutions, each containing 250 mg ZnCl_2_, were respectively added into the five kinds of WG solutions; and subsequently, the pH value of the five WG solutions was adjusted to 8–8.9 using 1 mol/L NaOH aqueous solution. And thus the mixed solutions were rested for 60 min, at a 37 °C water bath, until the self-assembled particles were completely precipitated. Lastly, the mixed suspension was dialyzed to neutrality in 60% ethanol, using a dialysis membrane (molecular weight cutoff of 12,000 Da), in which the retained precipitates were transferred to a Petri dish and freeze-dried at – 40 °C in a freeze dryer (SCIENTZ-10 N, Ningbo Xinzhi Biotechnology Co., Ltd.) for 24 h, to obtain the hybrid nanospheres.

### Property characterization

The XRD spectrum of the dried nanosphere sample was measured by X-ray diffractometer: line source of Cu targeted Ka, the wavelength of 0.15406 nm, scanning step length of 0.03°, scanning range of 10°–90°.

The infrared spectrum of the sample was measured by FTIR: The dried nanospheres and dried potassium bromide solid powder are mixed, grinded, and compressed to carry out a spectral scan.

The scanning wavenumber range was 4000–500 cm^−1^; after dispersing the prepared dried nanospheres on conductive tape sticked to the aluminum plate, and vacuuming, SEM was used to observe and take photos.

AAS was used to determine the Zn element content in the sample, and then calculate the ZnO content (3 parallel samples). A particle size analysis software of Nano Measurer was used to measure the average particle size of the nanospheres (number of samples 40), based on the SEM photos.

The specific surface and pore size analyzer were used to analyze the specific surface area and pore size of the sample (Using N_2_ as the adsorption medium): Before analysis, the sample was vacuum degassed at 50 °C for 3 h; the specific surface area of the sample was calculated by the Brunauer–Emmett–Teller (BET) model; the pore size distribution is calculated by the Barrett–Joyner–Halenda (BJH) model; the pore volume of micropores (diameter < 2 nm) is calculated by the HK/SF method; the pore volume of mesopores and macropores (diameter: 1.7–300 nm) is calculated by the BJH model.

### Evaluation of antimicrobial activity

Firstly the prepared ZnO/WG hybrid nanosphere sample (containing a ZnO mass ratio of 70.2%) and the positive control sample (commercially available nano-ZnO) were subjected to ultraviolet irradiation sterilization. Then, in the ultra-clean workbench with the cleanliness of class B, the dry particles of hybrid nanospheres were prepared into physiological saline suspensions of 10 mg/mL, 5 mg/mL, 2 mg/mL, 1 mg/mL, 0 mg/mL (respectively marked as a, b, c, d, e). After the activated *E. coli* and *S. aureus* (approximately 10^8^ Cfu/mL) were evenly coated on the agar solid medium (TSA) and standed still for 5 min until the bacterial suspension was absorbed by the agar plate, 25 µL each of the above four different concentrations of nanosphere suspensions, as well as a negative control sample, were dropped into the TSA medium previously coated with bacteria. After about 10 min later until the nanoparticle suspensions were absorbed by the agar plate, the cultures were inverted in a 32 °C microbial incubator and incubated for 24 h, finally to observe whether there is an inhibition zone at the drop sites, record the diameter (D) of the appeared inhibition zone.

### Determination of reactive oxygen species

Reactive oxygen species (ROS) were tested using DCFH-DA fluorescent probe kit. The ROS of the prepared ZnO/WG hybrid nanospheres were tested with PBS as the negative control and ZnO as the positive control. In brief, the testing procedures mainly included: reference substances and drugs were added to *S. aureus* bacterial solution of about 10^8^ CFU/mL, and cultured 60 min in an incubator at 37 °C, then the bacteria were centrifuged to remove the substances such as drugs and culture solution with acidic PBS lotion to obtain bacteria containing reactive oxygen species; subsequently, DCFH-DA fluorescent probe was added and cultured at 37 °C for 30 min, then the extracellular DCFG-DA was washed off with PBS, and the bacterium suspension was observed and photographed using an inverted fluorescence microscope.

### Safety evaluation

#### Hemolysis

1 mL of the whole blood of the mice was taken and put into a 2 mL centrifuge tube containing 2.5 μL of 2% heparin sodium and stirred slowly. Afterward, the same volume of saline solution was added and centrifuged at 1500 rpm for 10 min. The supernatant was removed and resuspended with 10 mL saline. The suspension was centrifuged at 1500 rpm for 20 min. The same process was repeated three times to obtain red blood cells^23^. Subsequently, 0.2 mL erythrocyte suspension was mixed with (I) 0.8 mL PBS as a negative control, (II) 0.8 mL deionized water as a positive control, and (III) 0.8 mL aqueous dispersion of ZnO/WG nanospheres (1–10 mg/mL). After incubation at 37 °C for 1 h and centrifuged at 12,000 rpm for 5 min, the supernatants were taken to determine the optical density (OD value) in a microplate reader (Spark 10 M, Tecan, Zürich, Switzerland) at a wavelength of 540 nm. The calculation formula of hemolysis ratio (Hr) is as follows: Hr (%) = (ODs − ODn/ODp − ODn) × 100%, in which ODs, ODp, and ODn are the OD values of the samples, the positive control, and the negative control, respectively.

#### Histological studies

To further evaluate the potential of ZnO/WG nanospheres in biomedical applications, we evaluated the possible biosafety and adverse reactions for 10 days. Female Balb/c mice were randomly divided into 2 groups (n = 3), and Intragastric administration (1) 0.9 wt% saline, (2) ZnO/WG nanospheres (10 mg/kg) every days. The body weight as an index of systemic toxicity was recorded and calculated every 2 days. After 10 days of treatment, major organs (including heart, liver, and kidney) were cut, half-sectioned, photographed and then placed in a buffered 4% formaldehyde solution overnight. Deparaffinized 5 μm sections were stained with hematoxylin–eosin for further analysis.

### Statistical analysis

Use SPSS Statistics 17.0 software to check all data and express them as mean ± standard deviation (SD). Use Student’s *t*-test to analyze statistical significance; *p < 0.05 was considered the minimum meaning.
